# Stimulation of osteoblast differentiation with guided ultrasound waves

**DOI:** 10.1186/s40349-015-0034-7

**Published:** 2015-08-05

**Authors:** James Macione, Daniel Long, Sterling Nesbitt, Scott Wentzell, Hiroki Yokota, Vaibhav Pandit, Shiva Kotha

**Affiliations:** Department of Biomedical Engineering, Rensselaer Polytechnic Institute, Troy, NY 12180 USA; Department of Biomedical Engineering, Indiana University Purdue University Indianapolis, Indianapolis, IN 46202 USA

## Abstract

**Background:**

Ultrasound induces mechanical vibration and heat, causing differentiation and proliferation in osteoblasts. All known *in vitro* evaluations of ultrasound are, however, performed with longitudinal ultrasound waves. We addressed a question: Do other forms of ultrasound waves, such as guided waves (longitudinal and guided flexural) transduced at a remote location, enhance differentiation of osteoblast cells?

**Methods:**

In this study, we employed guided Lamb waves that were induced in a borosilicate glass slide (cortical bone mimic). An average energy of 10–30 mW/cm^2^ for 20 min per day was applied to MC3T3 osteoblast-like cells, which were placed 30–75 mm distant from the transducer.

**Results:**

The result revealed that guided waves significantly stimulated the differentiation and mineralization of MC3T3 cells. In particular, guided waves elevated mRNA expression levels of bone formation-related genes such as alkaline phosphatase, osteopontin, osteocalcin, osteoprotegerin, and bone sialoprotein on days 8 and 16. In addition, the amount of mineralization found via Alizarin red staining was increased by 157 % (*p* = 0.034). The amount of mineralization was found to be independent of distance from the transducer (*p* = 0.967).

**Conclusion:**

We demonstrate herein that ultrasound in a form of guided Lamb waves is capable of inducing osteoblast differentiation *in vitro*, and it may enable the stimulation of osteoblasts *in vivo* over a distance from the site of ultrasound application.

## Background

Ultrasound can be used as a diagnostic and therapeutic tool in musculoskeletal diseases and disorders. As a diagnostic tool, for instance, it is utilized to evaluate mechanical properties of bone in which guided waves are induced into the cortical bone [1–4]. The induced waves are called “guided” as the cortex has a longitudinal speed of sound (SOS) and impedance that are double the surrounding tissue and internal trabecular bone [5]. As the guided wave propagates along the thin shelled cortex, particle motion-based flexing and extending occurs, heat is generated, and a small amount of mechanical energy, the leaky wave, is transmitted into the boundary tissues [6]. The concept of guided waves in the bone is not novel; commercial ultrasonic bone density devices utilize the guided waves over ranges from 5 to 8 cm [1, 2].

As a therapeutic tool, ultrasound in a form of longitudinal waves has been used to enhance bone growth and repair fracture including nonunion [7–12]. For example, ultrasound stimulates pre-osteoblasts to differentiate into osteoblasts [13–15]. The gold standard ultrasound signal is a 1.5-MHz longitudinal wave with a 20 % duty cycle that yields a spatial average, temporal average (SATA) intensity of 30 mW/cm^2^, and an FDA-approved power level [16]. The bone is treated via longitudinal waves, allowing waves to travel through soft tissue and ultimately reach the outer cortical surface. Sometimes, the waves can have conversion to shear and longitudinal components [16], even when induced at an angle to try to isolate these wave components [17].

The ultrasonic stimulation of the bone would serve a potential way to offset osteoporosis, but yet, it is only utilized for bone union and in culture [16, 17]. The reason for the limitations is because it has limited efficacy in stimulation within intact bone *in vivo*. Yet, these bone cells are sensitive to mechanical forces as the lack of this stimulation is one of the major causes of low bone mass. In an effort to understand why ultrasound can stimulate cells in culture, but not *in vivo*, some have started to examine different types of ultrasonic effects found in culture wells [18–20]. Others have evaluated different types of mechanical effects in culture such as frequency mixing [21] or variations of the applied frequency and intensity [22].

To our knowledge, all known *in vitro* evaluations of ultrasound stimulation of bone cells have been initiated with longitudinal waves passing through a medium prior to reaching the bone cells. This is not necessarily a configuration that will model what can occur *in vivo*. In this study, we explore the use of guided waves through a stimulated cortex in an effort to stimulate *in vitro* cells with guided Lamb waves.

## Materials and methods

### Guided wave speed of sound and wavelength in glass and bone

**Fig. 1:**
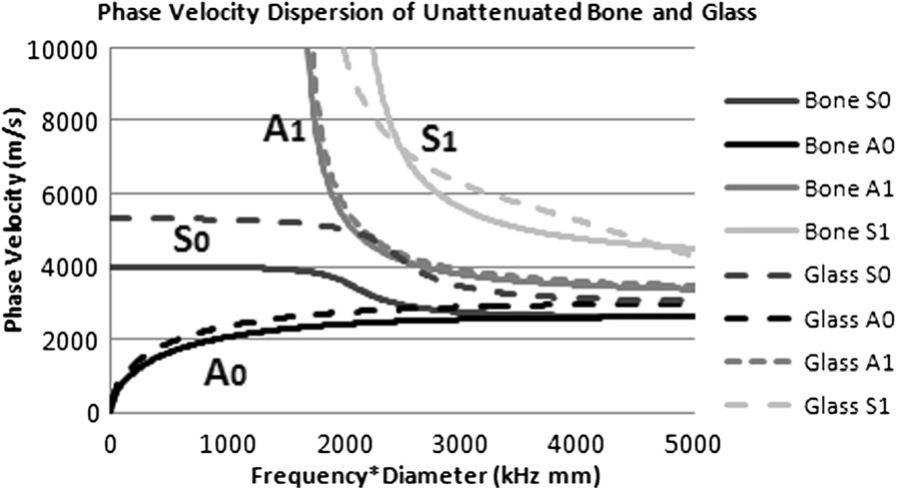
Lamb’s equations allow dispersion curves to be calculated which predict the speed of sound and modes which can be induced as a function that is the product of frequency and cortical thickness

The velocity of the different types of guided waves is dependent on the properties of the bone which include the thickness and longitudinal and transverse speed of sound. In order to compare the properties of flexural guided waves in glass and bone, Lamb’s equations were solved without fluid/tissue loading conditions using numerical methods on formulas with known constants from Dodd et al. [4] with methods from Rose [23] (Fig. 1). The phase velocity dispersion curve of the two lowest order antisymmetric and symmetric modes (*S*_0_, *S*_1_, *A*_0_, *A*_1_) for bone and borasilicate glass is shown in Fig. 1. In both dispersion curves, at a frequency*diameter (diameter of the cortical thickness) product of 1.2 MHz mm, there are two possible modes, the first-order antisymmetric (*A*_0_) and symmetric (*S*_0_).

### Cell culture and mineralization

Mouse osteoblast-like cells (MC3T3-E1 subclone 14, passage 7) were cultured in a solution of α-Modified Essential Medium (Invitrogen α-MEM 11900–024 with 2.2 g of NaHCO_3_), 10 % fetal bovine serum, and 100 μL/mL of penicillin/streptomycin [24]. The cells were grown in a T-75 culture flask until confluence was reached, at which point they were seeded into 8-well Lab-Tek chamber slides (catalog: 154534/glass, Thermofisher Scientific, Waltham, MA, USA) at a density of 4445 cells per 0.8 cm^2^. Differentiation was induced after confluence by adding mineralizing media. This mineralizing media is the aforementioned solution, supplemented with 10 mM β-glycerophosphate and 50 μg/mL of ascorbic acid. The media was changed the day after seeding and every 2 days thereafter. Note that when the media was changed, a heating pad was used to keep the cells warm (37 °C) since they were on a large glass sheet (75 × 100 mm) which induced a large, rapid drop in temperature (4–5 °C in 20 s) when placed in contact with the metal working surface of the biosafety cabinet.

### Slide preparation

Prior to attachment of the cell chambers, each borosilicate slide (product ID: 260230, Ted Pella, Redding, CA, USA) was washed and rinsed five times with soap/water and then rinsed for additional five times with deionized water. After drying, the slides were acid etched in piranha solution (3:1 ratio of concentrated sulfuric acid to 30 % hydrogen peroxide) in order to remove organic contaminants. The slides were then rinsed for additional five times in water before drying for 7 days in a biosafety cabinet under UV light (to assist with sterilization). After drying was complete, a Lab-Tek chamber slide was removed (by prying) from its glass slide and reseated onto the borosilicate glass. In order to prevent leaks, the edges of the chamber slides were reinforced by adding a small amount of PDMS (Sylgard 184 Kit, Dow Corning, Midland, Michigan) in 10:1 ratio (monomer:activator), which was cured overnight at 50 °C.

### Distance and attenuation effects

The use of chamber slides allows the effects of attenuation to be evaluated. The Lab-Tek chamber slides have a 2 × 4 well configuration in which each of the four groups of two are at different distances (30, 45, 60, and 75 mm) from the transducer. This 15-mm distance is large enough that attenuation-based changes in intensity can be measured at the four locations and then correlated with the amount of mineralization that occurs.

### Ultrasonic configuration

**Fig. 2:**
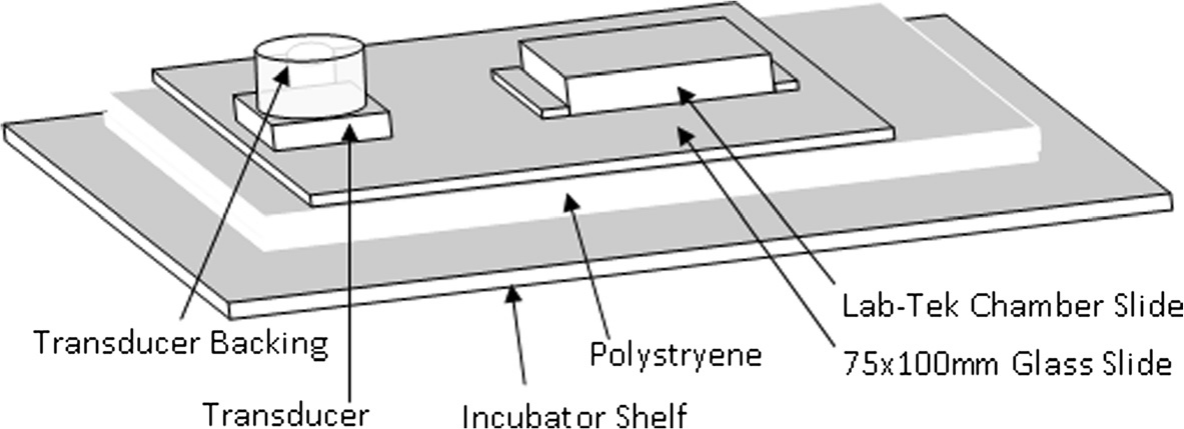
The cells are grown on an acid etched, optical grade borosilicate slide within the Lab-Tek Chamber slide. The distance between the chamber slide and the nearest edge of the transducer is approximately 30 mm. Lamb waves propagate through the glass which extend/flex it and allow ultrasound to “leak” out to stimulate the cells

Pulsed, guided waves were created so they would utilize the glass slide as a waveguide. An electronic timing circuit was used to pulse a 1-MHz ultrasound power amplifier at 1 KHz (SATA 30 mW) with 20 % duty cycle. The use of the pulsing circuitry allows comparison with other studies, as well as gives the slower modes (such as flexural *A*_0_) time to propagate though the plate (without being overtaken by faster modes), and also helps reduce the amount of standing waves. The borosilicate glass slide (75 × 10 × 1.2 mm^3^) was configured to act as a waveguide (Fig. 2) as the top was bound by cell media (*V*_L_ = 5600 vs. 1500 m/s) while the bottom was isolated from the metal trays of the incubator with a small sheet of 25-mm-thick expanded polystyrene. Thus, the pulsed guided waves would travel through the glass and leak into the cell chambers. Other losses would be governed by only attenuation and destructive interference.

### Validating guided and leaky waves

**Fig. 3:**
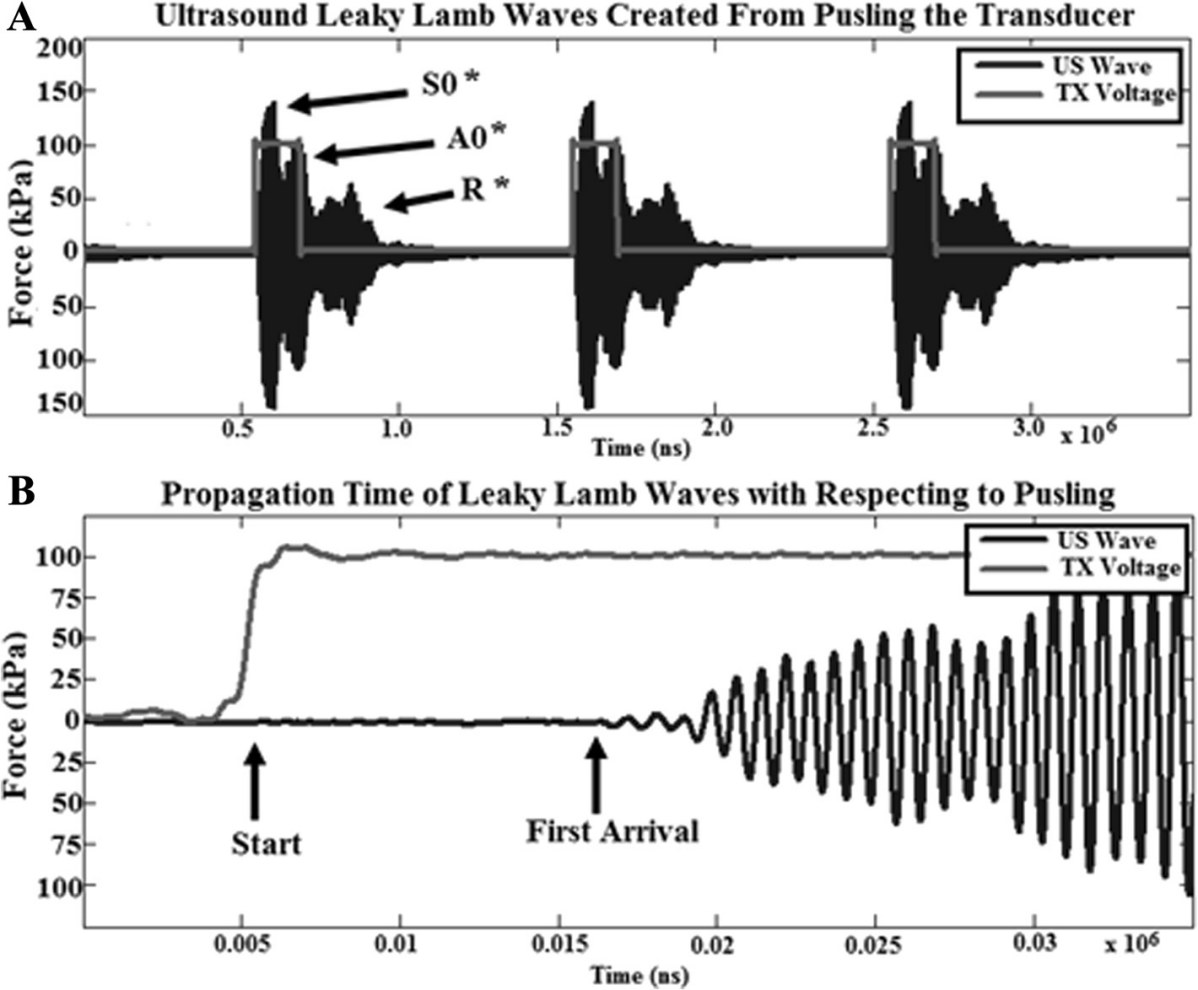
**a** A 1kHz drive signal with 20 % duty cycle plotted with the hydrophone recording from the chamber slide on the borosilicate plate. The *asterisk* indicates that the *S*_0_ and *A*_0_ modes are mixed since each transducer pulse will create both modes which have different SOS. **b** The first arrival can be used to compute the SOS in order to verify the presence of Lamb waves. In this case, over 75 mm, a SOS of 4900 m/s is determined which corresponds to the lowest order symmetrical Lamb mode (*S*_0_)

The presence of the guided waves as well as power of the leaky waves was validated by speed of sound measurements. A hydrophone was placed into a well filled with water. This signal, along with the start of the 1 kHz pulse was recorded by a 400-MHz data acquisition device (DR200 with PX14400, Signatec, Newport Beach, CA, USA). The first wave to arrive has a SOS of approximately 4800 m/s with the second group(s) at 2200 m/s, with some signals arriving later due to reflection (R) from the back of the glass plate (Fig. 3a). The first group of waves are denoted *S*_0_*, which indicates that it starts with the faster *S*_0_ waves but afterward becomes a combination of both modes. In the same manner, the slowest waves at the end of each pulsing cycle are denoted *A*_0_*. The waves between the start of *A*_0_* and end of *S*_0_* are a combination/superposition of both modes.

### Leaky lamb wave force

The amount of energy from the leaky waves used to stimulate the cells was measured with the HNR-1000 hydrophone after each well was filled with media. The hydrophone was placed in each well and slightly angled toward the transducer in order to find the peak intensity. The maximum peak voltage of the waveform over 30 s was converted to a power level (W/cm^2^) from the hydrophone calibration curve (V^2^ cm^2^/W) at 1 MHz and was found to vary from 30 to 10 mW/cm^2^ SATA from the well closest to the well furthest from the transducer.

### Alizarin red staining for calcium

Alizarin red staining was used to image and quantify mineralization [21]. Samples were fixed using 70 % ethanol for 1 h before mixing with 40 mM alizarin red at pH 4.2 for 10 min and finally washed with tap water before being allowed to dry. Mineralization of the samples was done via microscopy. The amount of calcium was then quantified by destaining with a solution of 10 % cetylpyridinium chloride and 10 mM sodium phosphate for 15 min at room temperature. The absorbance of each sample was determined at 562 nm and calibrated to standards of alizarin red diluted with 10 mM sodium phosphate containing 10 % cetylpyridinium chloride. In order to determine statistical relevance, a post hoc Tukey and paired *t* tests Minitab 15 (State College, PA) were used for ANOVA analysis. Statistical significance is considered any *p* < 0.05. A total of eight glass slides were used to access mineralization, this yielding eight wells at each distance (two wells at each distance on one plate) from the transducer for both the control and ultrasound stimulated samples.

### Quantitative polymerase chain reaction

Quantitative PCR (qPCR) was performed to analyze mRNA expression levels that indicate cellular differentiation. Total RNA was extracted with TRIzol LS reagent (Life Technologies, Carlsbad, CA, USA) and treated with deoxyribonuclease. After DNase treatment, reverse-transcription was conducted using oligo-p(dT) primers. PCR amplification was performed on Lightcycler 480 (Roche, Penzberg, Germany) using SYBR green master mix (Qiagen, Venlo, Netherlands) and PCR primers in Table 1. Reactions were carried out in total volumes of 20 μL and included 0.3 μM of each primer (forward and reverse) and 0.5 μL of diluted cDNA template containing 1000 ng of cDNA. Statistical significance was determined with the MATLAB (Mathworks, Natick, MA, USA) ANOVA function. Statistical significance is considered any *p* < 0.05. A total of eight glass slides (four control, four stimulated) were used for PCR, but due to the expense of running reactions, the wells on each were mixed to form a single sample.

**Table 1:** Primers for qPCR in this study

Gene	Forward primer	Reverse primer
GAPDH	AACGACCCCTTCATTGAC	TCCACGACATACTCAGCAC
OPN	GATCAGGACAACAACGGAAAGG	CTTGTGGCTGTGAAACTTGTGG
OCN	AGGGAGGATCAAGTCCCG	GAACAGACTCCGGCGCTA
BSP	TGTCTGCTGAAACCCGTTC	GGGGTCTTTAAGTACCGGC
ALP	GTTGCCAAGCTGGGAAGAACAC	CCCACCCCGCTATTCCAAAC
OPG	CGAGGACCACAATGAACAAGTG	TTTTAGGTAGGTGCCAGGAGCA

*GAPDH* glyceraldehyde-3-phosphate dehydrogenase, *OPN* osteopontin, *OCN* osteocalcin, *BSP* bone sialoprotein, *ALP* alkaline phosphatase, and *OPG* osteoprotegerin

## Results

**Fig. 4:**
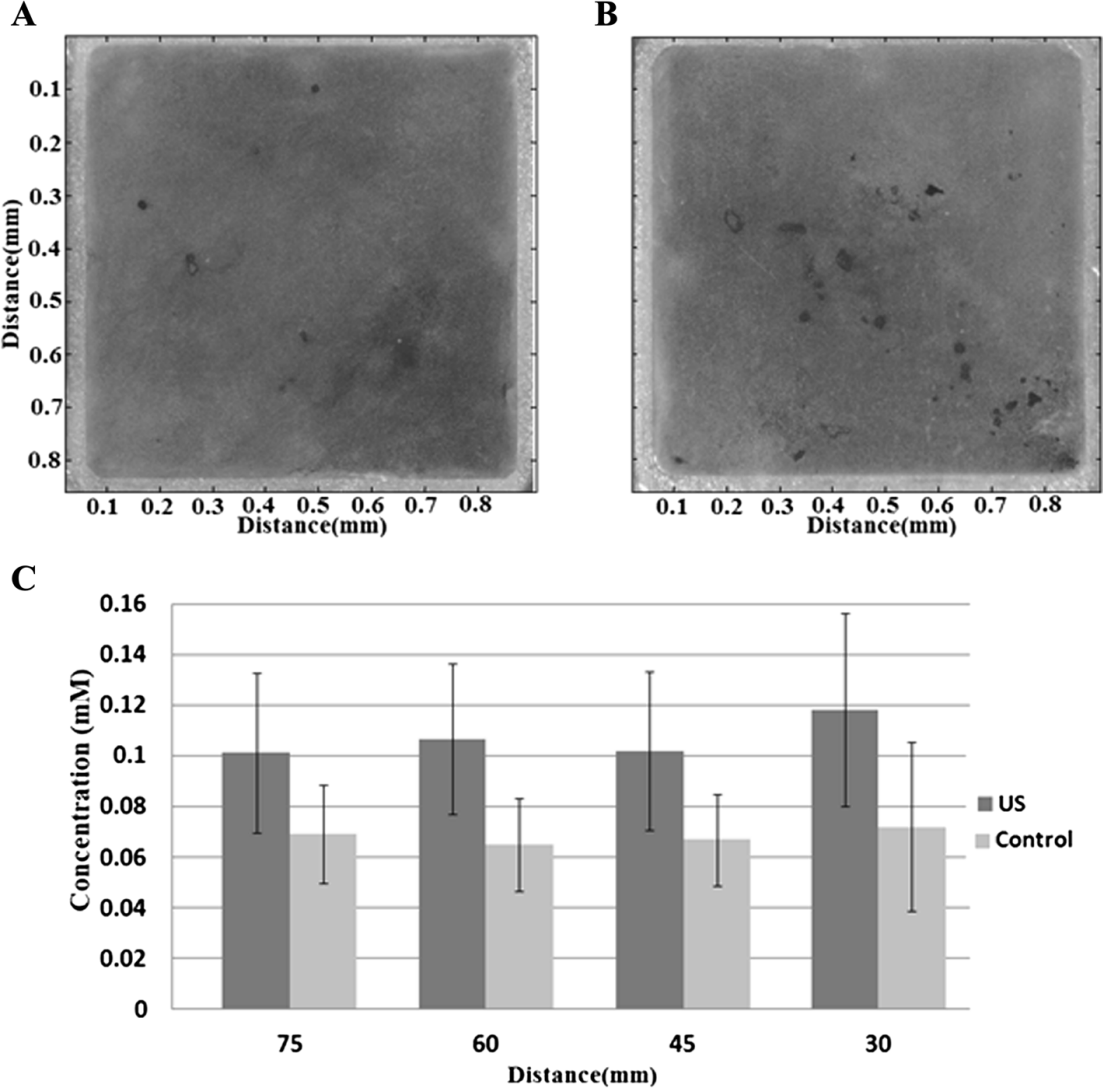
**a**, **b** Representative images from alizarin red staining of both the control (**a**) and guided wave samples (**b**) at day 25. Quantifying the mineralizaiton (**c**) demonstrates that samples stimulated with guided waves have an average of 157 % more calcium, an effect which was independent of distance

Alizarin red staining demonstrated that both the control and ultrasound-stimulated sample had nodule formation. However, the mineral deposition was increased in the samples stimulated by guided waves (Fig. 4a–c). Quantification of mineralization through a process of Alizarin red destaining revealed that an average of 157 % more mineralization was achieved in the stimulated samples (*p* = 0.034).

The increase in mineralization was not dependent on the distance from the transducer (*p* = 0.967). This distance from the transducer is related to the amount of leaky wave energy the cells would experience, ranging from 30 mW/cm^2^ at 30 mm to 10 mW/cm^2^ at 75 mm. There was a slightly higher mineralization at the 30-mm sample, but the difference among the samples was not statistically significant.

### Guided wave-induced elevation of relative mRNA expression levels

The relative mRNA expression levels of the five selected genes were examined on days 8 and 16 in the presence and absence of ultrasound in guided waves. At both time points, the relative expression levels were significantly greater than 1, indicating that differentiation of osteoblasts was accelerated in the ultrasound-stimulated samples (Table 2). At day 8, the relative gene levels for alkaline phosphatase (ALP), osteopontin (OPN), osteocalcin (OCN), osteoprotegerin (OPG), and bone sialoprotein (BSP) increased 2.5–6.8-fold. The relative gene expression became larger at day 16, except for ALP and OPG. For instance, the relative mRNA level of BSP was 6.8 ± 1.1 (mean ± s.d.; *p* < 0.01) on day 8 and 10.2 ± 4.2 (*p* < 0.01) on day 16. The decrease in ALP is expected since it is considered as an early marker for osteoblast differentiation [25].

**Table 2:** Fold change in relative mRNA levels by guided waves

	Day 8		Day 16	
	Mean ± s.d.	*p* value	Mean ± s.d.	*p* value
OPN	4.2 ± 0.8	0.03	5.1 ± 1.0	0.00
OCN	2.5 ± 2.2	0.36	6.9 ± 3.4	0.00
ALP	3.8 ± 2.5	0.01	2.3 ± 0.5	0.00
BSP	6.8 ± 1.1	0.00	10.2 ± 4.1	0.00
OPG	3.1 ± 0.42	0.00	1.5 ± 0.4	0.04

## Discussion

Guided waves travel within cortical bone and can have longitudinal and Lamb wave components. The guided waves will leak energy into tissue boundaries and in the case of Lamb waves also can create very small amounts of flexion from out-of-plane particle motion. In this study, we created an acoustic representation of cortical bone with glass (Fig. 2) and then induced 1.2-MHz-mm guided flexural waves (Fig. 1) through a 30-mm path length (Fig. 3) to produce a leaky wave SATA of 10 to 30 mW/cm^2^. The cells were thus stimulated by leaky energy from the flexural waves which originate from the plate on which they reside as well as any small amounts of flexion which may occur. This is a different mechanism from the current standard of ultrasonic stimulation, in which only those cells directly within the field of the transducer are stimulated via the mechanism of longitudinal waves.

The guided waves were found to stimulate differentiation and mineralization in osteoblasts. Relative mRNA expression levels of the selected bone-linked markers increased three- to fivefold in 8 days of the ultrasound stimulation (Table 2). The mRNA levels stayed elevated on day 16, thus showing a continuous response to treatment. The guided waves also induced more mineralization, which could be seen in the form of nodules (Fig. 4b, d) which translated to greater calcium concentration (Fig. 4e). This increased mineralization was not found to be dependent on the distance of the sample to the transducer even though the leaky power from the Lamb waves was attenuated (30 to 10 mW/cm^2^).

There was no dependence on the amount of mineralization with the distance from the transducer (leaky wave power). It is likely that the difference in intensity levels from 10 to 30 mW/cm^2^ was not large enough to create a statically significant response. The results are consistent with a previous study that found that the cells stimulated with smaller (20 mW/cm^2^) range of power level show only a small difference in response [22].

There were limitations to this study which were related to the selection of ultrasonic parameters. Ultrasound values of the driving frequency, power level, and pulse repetition frequency were selected by what others have done with longitudinal waves, and no attempt was made to optimize them for guided waves. For example, the pulsing frequency will alter the superposition (destructive interference of slower modes with faster) of the bulk of the pulses and even alter the amount of standing waves. The thickness of the glass was also a limitation in that it needed to be thin enough to allow light microscopy at the expense of mode velocity.

## Conclusion

In this study, a mechanism of generating an osteogenic response was evaluated *in vitro*. Pulsed guided waves were found to produce a positive response when compared to a control sample (unstimulated). The guided waves allow cells distant from the transducer to be stimulated by allowing the energy to travel through the bone. Further work would be needed to optimize ultrasonic parameters specific to guided waves. Also, more knowledge related to the physics of guided flexural waves would be of interest.

## Figures and Tables

**Fig. 5:**
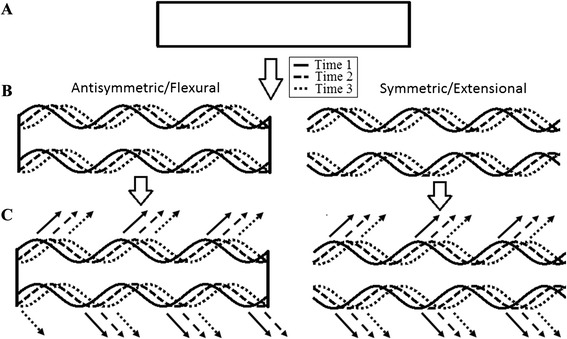
**a** A schematic of a short section of a plate. **b** Exaggerated symmetric and antisymmetic flexural waves propagating through a plate at three closely related time points. **c** Both types of Lamb waves produce leaky waves which propagate out of the medium with respective directions. The symmetric half of the figure is from Lee and Kuo [6]

## Appendix

### Calculation of the Lamb mode velocity

The velocity of each of the Lamb modes in glass were computed from dispersion curves which are dependent on properties of the bone including the longitudinal (*V*_L_) and transverse (*V*_s_) SOS and *d*. The velocity dispersion of the Lamb waves are described with the characteristic Eqs. 1, 2, 3, 4, 5, and 6 [23]. The wave number *k*_SA_ is numerically equal to *ω*/*V*, where *V* is the phase velocity of the Lamb wave and *ω* is the angular frequency. The phase velocity (*V*) is related to the *λ* by the relationship *V* = (*ω*/2*π*) *λ* [23]. Thus, Eqs. 1, 2, 3, 4, 5, and 6 were solved numerically with Newton-Raphson technique to get the dispersion curves through glass:

1 $$ {\left({k_{\mathrm{SA}}}^2+{T_{\mathrm{SA}}}^2\right)}^2 \coth \left({L}_{\mathrm{SA}}d\right)-4{k_{\mathrm{SA}}}^2{L}_{\mathrm{SA}}{T}_{\mathrm{SA}} \coth \left({T}_{\mathrm{SA}}d\right)=0,\ \mathrm{Symmetric} $$

2 $$ {\left({k_{\mathrm{SA}}}^2+{T_{\mathrm{SA}}}^2\right)}^2 \tanh \left({L}_{\mathrm{SA}}d\right)\hbox{-} 4{k_{\mathrm{SA}}}^2{L}_{\mathrm{SA}}{T}_{\mathrm{SA}} \tanh \left({T}_{\mathrm{SA}}d\right)=0,\ \mathrm{Antisymmetric} $$

where

3 $$ {L}_{\mathrm{SA}}=\sqrt{{k_{\mathrm{SA}}}^2\hbox{-} \frac{w^2}{\mathrm{Vl}}} $$

4 $$ {T}_{\mathrm{SA}}=\sqrt{{k_{\mathrm{SA}}}^2\hbox{-} \frac{w^2}{\mathrm{Vs}}} $$

5 $$ {Q}_{\mathrm{SA}}=\sqrt{{k_{\mathrm{SA}}}^2\hbox{-} \frac{w^2}{\mathrm{Vl}}} $$

6 $$ {K}_T=\frac{w}{\mathrm{Vs}} $$

As the Lamb wave propagates, at a level of particle motion, there is flexion around its midline and allows energy to escape (Fig. 5). The flexion and extension create different types of Lamb waves which are classified as antisymmetric and symmetric modes, respectively. The wave motions of the antisymmetic and symmetric waves have different propagation velocities, a property that depends on *d* as well as the transducer frequency used to induce it.
